# The Contact Properties of Monolayer and Multilayer MoS_2_-Metal van der Waals Interfaces

**DOI:** 10.3390/nano14131075

**Published:** 2024-06-24

**Authors:** Xin Pei, Xiaohui Hu, Tao Xu, Litao Sun

**Affiliations:** 1 College of Materials Science and Engineering, Nanjing Tech University, Nanjing 211816, China; 202161103005@njtech.edu.cn; 2 Jiangsu Collaborative Innovation Center for Advanced Inorganic Function Composites, Nanjing Tech University, Nanjing 211816, China; 3 SEU-FEI Nano-Pico Center, Key Laboratory of MEMS of Ministry of Education, Southeast University, Nanjing 210096, China; xt@seu.edu.cn (T.X.); slt@seu.edu.cn (L.S.)

**Keywords:** two-dimensional materials, transition metal dichalcogenides, electrical properties, Schottky barrier, density functional theory

## Abstract

The contact resistance formed between MoS_2_ and metal electrodes plays a key role in MoS_2_-based electronic devices. The Schottky barrier height (SBH) is a crucial parameter for determining the contact resistance. However, the SBH is difficult to modulate because of the strong Fermi-level pinning (FLP) at MoS_2_-metal interfaces. Here, we investigate the FLP effect and the contact types of monolayer and multilayer MoS_2_-metal van der Waals (vdW) interfaces using density functional theory (DFT) calculations based on Perdew–Burke–Ernzerhof (PBE) level. It has been demonstrated that, compared with monolayer MoS_2_-metal close interfaces, the FLP effect can be significantly reduced in monolayer MoS_2_-metal vdW interfaces. Furthermore, as the layer number of MoS_2_ increases from 1L to 4L, the FLP effect is first weakened and then increased, which can be attributed to the charge redistribution at the MoS_2_-metal and MoS_2_-MoS_2_ interfaces. In addition, the p-type Schottky contact can be achieved in 1L–4L MoS_2_-Pt, 3L MoS_2_-Au, and 2L–3L MoS_2_-Pd vdW interfaces, which is useful for realizing complementary metal oxide semiconductor (CMOS) logic circuits. These findings indicated that the FLP and contact types can be effectively modulated at MoS_2_-metal vdW interfaces by selecting the layer number of MoS_2_.

## 1. Introduction

In recent years, two-dimensional (2D) transition metal dichalcogenides (TMDCs) have received much attention due to their potential application in high-performance and low power devices [1,2,3,4,5,6,7,8]. Among the TMDCs, MoS_2_ is considered as the most potentially useful material because of its suitable band gap, high on/off ratio, and high room temperature carrier mobility [9,10,11]. Although MoS_2_ exhibits the most promising properties, the high contact resistance between MoS_2_ and metal electrodes severely limits MoS_2_-based device performance [12,13,14,15].

The Schottky barrier height (SBH) serves as a crucial parameter for contact resistance in a metal-semiconductor contact, which essentially determines the efficiency of charge transport and has a significant influence on device performance [16]. According to the Schottky–Mott rule, we can modulate the SBH by using metal electrodes with different work functions and obtain low contact resistance. However, the SBH actually exhibits a weak dependency on metal work functions due to the strong Fermi-level pinning (FLP) effect [17,18,19]. The pinning factor S, ranging from 0 to 1, is an indicator used for evaluating the strength of FLP effect. S = 1 suggests no pinning at the metal-semiconductor interface, whereas S approaching 0 implies that the FLP is getting stronger. For instance, although metals with high work functions (such as Au and Pd) are used as electrodes, the MoS_2_-based field effect transistors (FETs) always exhibit n-type Schottky contacts due to the strong FLP effect [20]. Therefore, reducing the FLP effect is significantly important for achieving the tunable SBH and thus creating high-performance electrical devices.

Commonly, the strong interactions at the metal-semiconductor interfaces lead to the localized density of states, which will induce the FLP effect. Metal-induced gap states (MIGS) [21] and defect/disorder-induced gap states (DIGS) [22] are important factors for contributing to the FLP effect. The DIGS can be neglected at the high-quality metal-semiconductor interfaces. Additionally, the interface dipole is another factor that contributes to the FLP effect, which is induced by the redistribution of charge at the metal-semiconductor interfaces [23,24]. Thus, reducing the MIGS and the interface dipole is important for weakening the FLP effect.

Up to date, many strategies have been adopted to reduce the FLP effect, such as utilizing edge contact [25,26], inserting buffer layers [27,28], and using 2D metals as electrodes [11,13,14,29,30]. Adopting the edge-contact strategy, Yang et al. [26] successfully weakened the FLP effect between Pd and MoS_2_, thereby achieving an S value of 0.975, obeying the Schottky–Mott rule. The transition metal oxides (TMOs) have been used as insertion materials for reducing the FLP effect [28]. A reduced FLP effect and tunable SBH can be achieved in CrX_3_ (X = I, Br)/2D metal contacts [29]. However, the edge-etching process is complicated, and inserting buffer layers is often hindered by the deposition conditions [31].

Compared to the above-mentioned strategies, the van der Waals (vdW) contact between 2D semiconductors and bulk metals presents superiority for reducing the FLP effect because the vdW contact will create the ultraclean surface without damaging the structure of 2D materials [32,33,34,35,36]. Compared with traditional metal-deposition techniques [37,38] used for constructing close interfaces, the low-energy vdW integration process physically laminates the prefabricated metal electrodes onto the MoS_2_, resulting in atomically clean vdW interfaces [33,34]. Kong et al. [32] demonstrated that the vdW integration process does not impose strains or doping effects on the 2D semiconductor. Duan et al. [33] indicated that vdW integration enables efficient electron tunneling in 2D MoS_2_, demonstrating its potential for fabricating superlattices or artificial heterostructures. Liu et al. [34] reported that the use of a low-energy vdW metal-integration technique enables the creation of a MoS_2_ vertical FET with an on/off ratio of 10^3^, which is related to the high-quality metal-semiconductor interface. Wang et al. [36] reported the realization of vdW contacts between In-Au alloys and monolayer MoS_2_, which achieved low-resistance contacts with excellent performance. Liu et al. [39] achieved the vdW contacts between MoS_2_ and 3D metals using a transfer-metal method, and they obtained an FLP factor of S = 0.96, which is very close to the Schottky–Mott limit. Although the FLP factor close to the Schottky–Mott limit has been found in MoS_2_-metal vdW interfaces, the mechanism underlying the FLP effect is incompletely known.

On the other hand, the band structure of MoS_2_ is dependent on the layer number and thus influences the contact properties [40,41,42,43,44]. For example, Kou et al. [42] showed that the electronic band structure of TMDC heterostructures have a sensitive dependence on their relative thickness. Cui et al. [43] illustrated that the carrier mobility of MoS_2_-based devices can be improved by increasing the layer number of MoS_2_. Lee et al. [44] demonstrated that an extremely low SBH of 70 meV can be achieved at the Al-MoS_2_ interface using trilayer MoS_2_. Therefore, the thickness of MoS_2_ is also a crucial parameter for influencing the SBH and the contact resistance.

In this work, based on the density functional theory (DFT), we investigate the FLP effect and the contact types of monolayer and multilayer MoS_2_-metal vdW interfaces. Compared to monolayer MoS_2_-metal close interfaces, the FLP effect is obviously reduced in monolayer MoS_2_-metal vdW interfaces, which can be attributed to the weak MIGS and small interface dipoles at vdW interfaces. Furthermore, we found that the FLP effect in multilayer MoS_2_-metal vdW interfaces is dependent on the layer number of MoS_2_. Due to the weak FLP, the p-type Schottky contact can be achieved for high-work-function metals such as 1L–4L MoS_2_-Pt, 3L MoS_2_-Au, and 2L–3L MoS_2_-Pd vdW interfaces. These findings provide an effective method for reducing the FLP effect and thus facilitating the development of high-performance MoS_2_-based devices.

## 2. Computational Methods

The DFT calculations are carried out using the Vienna Ab initio Simulation Package (VASP) [45,46]. The projector-augmented wave (PAW) [47] potentials are used to treat the electron–ion interaction. To describe the exchange-correlation interaction, the Perdew–Burke–Ernzerhof (PBE) formulation of the generalized gradient approximation (GGA) [48] is adopted. The vdW interaction between MoS_2_ and metals is treated using the DFT-D3 approach within the Grimme scheme [49]. A plane-wave cutoff energy of 500 eV is used. The Brillouin-zone integration is performed using a 11 × 11 × 1 k-mesh. The energy convergence criterion is 10^−5^ eV and the force convergence criterion is 0.01 eV Å^−1^. A vacuum region of 18 Å in the z direction is used to eliminate the interaction between the neighboring slabs.

## 3. Results and Discussion

### 3.1. Model Structures

The optimized lattice parameter of monolayer (1L) MoS_2_ is 3.19 Å and the bandgap of 1L MoS_2_ is 1.64 eV, which is in agreement with previous studies [11,50]. We construct the metal surfaces using six layers of metal atoms (Al, Ag, Cu, Au, Pd, and Pt in (111) orientation). The work functions of these metals are in the range of 4.15–5.65 eV, as listed in Table 1, in agreement with the previous results [51,52]. MoS_2_-metal-close and -vdW interfaces are constructed by vertically stacking MoS_2_ and metal surfaces. The lattice parameter of MoS_2_ is fixed and the metal’s lattice constant is strained to match that of MoS_2_. The supercell match patterns are ($\sqrt{3}\;$×$\;\sqrt{3}$) R30° MoS_2_/(2 × 2) metal surfaces. It can be seen from Table 1 that the lattice mismatches between MoS_2_ and metal surfaces (Al, Ag, Au, Pd, and Pt in (111)) ranged from 0.42% to 4.57%. In contrast, for MoS_2_/Cu (111) surface the lattice mismatch is 6.94%.

Considering the relatively large lattice mismatch between MoS_2_ and Cu (111) surface, we also examine the supercell match of (4 × 4) MoS_2_/(5 × 5) Cu, which corresponds to the small lattice mismatch (0.71%). Taking a 1L MoS_2_-Cu close interface as example, we calculate its projected band structures, as shown in Figure S1 of supporting information. It can be found that the n-SBH changes slightly from 0.18 eV to 0.21 eV for MoS_2_-Cu close interfaces. The calculated result demonstrates that, compared with the lattice mismatch of 6.94%, the small lattice mismatch (0.71%) has a negligible influence on the SBH value of the 1L MoS_2_-Cu close interface. Meanwhile, given that the high computational cost of multilayer MoS_2_, the supercell match of ($\sqrt{3}\;$×$\;\sqrt{3}$) R30° MoS_2_/(2 × 2) Cu (111) is adopted in the following procedures.

**Figure 1:**
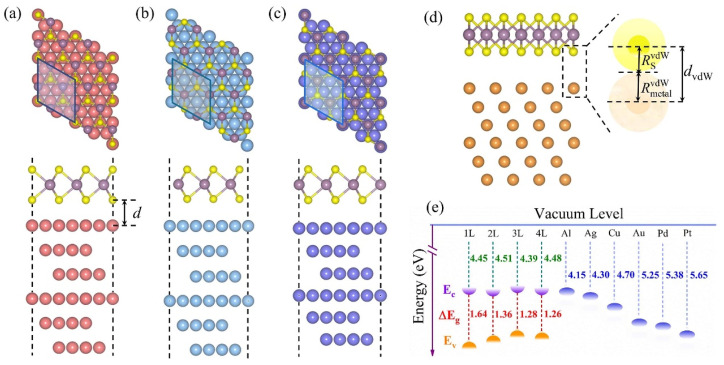
The top and side views for (**a**) 1L MoS_2_-Al/Pt, (**b**) 1L MoS_2_-Ag/Cu/Au, and (**c**) 1L MoS_2_-Pd. (**d**) Schematic illustration of the interlayer distance (*d*_vdW_); $R_{S}^{\mathrm{vdW}}$ and $R_{\mathrm{metal}}^{\mathrm{vdW}}$ are the vdW radii of S atoms and metal atoms, respectively. (**e**) The band alignments of MoS_2_ and metals. E_c_, E_v_, and ΔE_g_ represent the conduction band edge, valence band edge. and band gap of MoS_2_, respectively.

After structural optimization, we obtained the most stable structures of 1L MoS_2_-metal close interfaces, as shown in Figure 1a–c. As for MoS_2_-Al (Pt), Mo atoms are located above the centers of the triangles formed by the top, fcc, and hcp positions, and S atoms are located above the metal atoms, as presented in Figure 1a. On Ag, Cu, and Au (111) surfaces, Mo and S atoms are all located above the centers of the triangles formed by the top, fcc, and hcp positions (Figure 1b). In the case of MoS_2_-Pd, Mo atoms are located above the metal atoms, and S atoms are located above the centers of the triangles formed by the top, fcc, and hcp positions, as displayed in Figure 1c. The initial structures of multilayer MoS_2_-metal close interfaces are constructed on the basis of the most stable 1L MoS_2_-metal close interfaces. For MoS_2_-metal vdW interfaces, the atomic stacking at MoS_2_ and metal interfaces is same as that of MoS_2_-metal close interfaces. The interlayer distance is different at MoS_2_-metal-close and -vdW interfaces.

The interlayer distance *d* is defined as the average distance between the S atoms and metal atoms closest to the interface in MoS_2_-metal close interfaces, as illustrated in Figure 1a. The optimized *d* values are in the range of 2.22–2.81 Å for MoS_2_-metal close interfaces, as listed in Table 1. As for MoS_2_-metal vdW interfaces, the interlayer distances *d*_vdW_ are set as ${d_{\mathrm{vdW}}=R}_{S}^{\mathrm{vdW}}+R_{\mathrm{metal}}^{\mathrm{vdW}}$, where $R_{S}^{\mathrm{vdW}}$ and $R_{\mathrm{metal}}^{\mathrm{vdW}}$ are the vdW radii of S atoms and metal atoms [53], respectively, as shown in Figure 1d. The calculated *d*_vdW_ values are between 3.46 and 3.60 Å (listed in Table 1), which is larger than that of close interfaces, implying a weak interaction at MoS_2_-metal vdW interfaces.

To examine the stability of 1L MoS_2_-metal-close and -vdW interfaces, we calculate their binding energies. The binding energies can be defined as $E_{b}=(E_{{\mathrm{MoS}}_{2}/\mathrm{metal}}-E_{{\mathrm{MoS}}_{2}}-E_{\mathrm{metal}})/N$, where $E_{{\mathrm{MoS}}_{2}/\mathrm{metal}}$, $E_{{\mathrm{MoS}}_{2}}$ and $E_{\mathrm{metal}}$ are the total energies of 1L MoS_2_-metal-close and -vdW interfaces, MoS_2_, and metal surfaces, respectively. N is the number of Mo atoms in 1L MoS_2_-metal-close and -vdW interfaces. According to the definition, the negative binding energy demonstrates that 1L MoS_2_-metal-close and -vdW interfaces are energetically stable. As listed in Table 1, for 1L MoS_2_-metal close interfaces the binding energies vary from −0.98 eV to −0.42 eV. In contrast, for 1L MoS_2_-metal vdW interfaces, the binding energies range from −0.43 eV to −0.29 eV. These results indicate that 1L MoS_2_-metal-close and -vdW interfaces are energetically favorable. In addition, we found that 1L MoS_2_-metal close interfaces have greater negative binding energies than those of the corresponding 1L MoS_2_-metal vdW interfaces, suggesting that 1L MoS_2_-metal close interfaces are more favorable than the equivalent vdW interfaces.

### 3.2. SBH of 1L MoS_2_-Metal-Close and -vdW Interfaces

**Figure 2:**
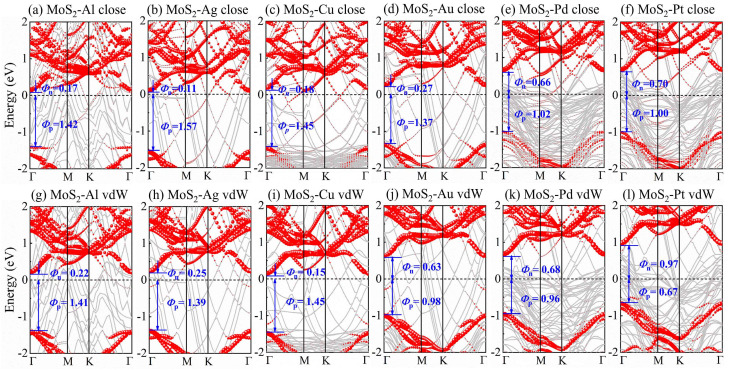
The projected band structures of (**a**–**f**) 1L MoS_2_-metal close interfaces and (**g**–**l**) 1L MoS_2_-metal vdW interfaces. The gray curves represent the band structures of MoS_2_-metal interfaces. The red-dotted curves denote the band structures of MoS_2_.

Based on the Schottky–Mott model [21], the n-type SBH (Φ_n_) and p-type SBH (Φ_p_) are defined as Φ_n_ = E_CBM_ − E_F_, Φ_p_ = E_F_ − E_VBM_, respectively. Where E_CBM_, E_F_, and E_VBM_ are the conduction band minimum (CBM), the Fermi energy, and the valence band maximum (VBM), respectively. The n-type and p-type SBHs can be extracted from the projected band structures of MoS_2_-metal-close and -vdW interfaces, which are illustrated in the upper and lower panels of Figure 2, respectively. It can be observed that the Fermi level is closer to that of the CBM, indicating that the n-type Schottky contacts are formed in all 1L MoS_2_-metal close interfaces due to the strong FLP, which are in agreement with previous reports [54,55]. For 1L MoS_2_-metal vdW interfaces, MoS_2_-Al (or Ag, Cu, Au, or Pd) still preserves the n-type Schottky contacts. Among them, MoS_2_-Al (or Ag or Cu) has a small n-SBH (0.15–0.25 eV), which ensures the low contact resistance that is observed experimentally [36]. However, the n-type Schottky contact transforms into the p-type Schottky contact in MoS_2_-Pt-vdW interfaces, suggesting that the FLP is reduced in 1L MoS_2_-metal vdW interfaces. Therefore, it is easier to achieve p-type Schottky contact at the vdW interface between MoS_2_ and high-work-function metals, which is useful for realizing complementary metal oxide semiconductor (CMOS) logic circuits [56].

### 3.3. FLP Strength of 1L MoS_2_-Metal-Close and -vdW Interfaces

**Figure 3:**
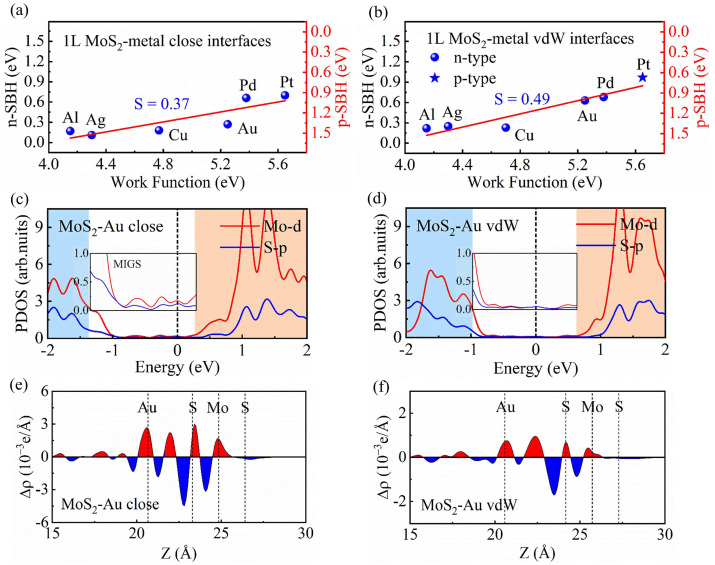
The SBHs versus the metal work functions for (**a**) 1L MoS_2_-metal close and (**b**) 1L MoS_2_-metal vdW interfaces. The partial density of states (PDOS) of (**c**) 1L MoS_2_-Au close and (**d**) 1L MoS_2_-Au vdW interfaces. The magnified PDOS represents the MIGS. The valence band is shaded in blue and the conduction band is shaded in orange. (**e**) The plane average charge-density difference Δ*ρ* (z) of 1L MoS_2_-Au close and (**f**) 1L MoS_2_-Au vdW interface. The red and blue colors represent charge accumulation and depletion, respectively.

To have a quantitative description of the FLP strength, we calculated the pinning factor S, which is defined as $S=d\Phi_{B}/dW_{M}$, where Φ_B_ represents the SBH and W_M_ denotes the metal work function. Based on the definition, S = 0 denotes a strong FLP in MoS_2_-metal-close and -vdW interfaces, whereas S = 1 represents the ideal Schottky–Mott limit. The S values of 1L MoS_2_-metal-close and -vdW interfaces are fitted in Figure 3a,b. It can be seen from Figure 3a that the pinning factor S = 0.37 for 1L MoS_2_-metal close interfaces, indicating a strong FLP effect at the interface, which is in agreement with previous results [17]. The pinning factor S of 1L MoS_2_-metal vdW interfaces is 0.49, which is much larger than that of the 1L MoS_2_-metal close interfaces, suggesting a weak FLP at the vdW interfaces.

To understand the weak FLP in 1L MoS_2_-metal vdW interfaces, taking 1L MoS_2_-Au as an example, the projected density of states (PDOS) of Mo and S atoms are calculated and displayed in Figure 3c,d. For a 1L MoS_2_-Au close interface, a large number of Mo-d and S-p states are extended to the forbidden band of MoS_2_, leading to the obvious MIGS that can be seen from the magnified PDOS inserted in Figure 3c. Compared with the case of the 1L MoS_2_-Au close interface, the MIGS are notably reduced in the 1L MoS_2_-Au vdW interface, as shown in Figure 3d. We also calculate the PDOS of 1L MoS_2_-Al (Ag, Cu, Pd and Pt) close and vdW interfaces, as shown in Figure S2 in the supporting information. We found that, similar to the case of the 1L MoS_2_-Au vdW interface, fewer MIGS are also found in 1L MoS_2_-Al (Ag, Cu, Pd and Pt) vdW interfaces.

The FLP strength can also be influenced by the interface dipole at MoS_2_-metal interfaces. Interface dipole formation is related to charge redistribution, which can be characterized from the charge density difference (Δ*ρ*) at MoS_2_-metal interfaces. The charge density difference is defined as $\Delta \rho =\rho_{{\mathrm{MoS}}_{2}/\mathrm{metal}}-\rho_{{\mathrm{MoS}}_{2}}-\rho_{\mathrm{metal}}$, where $\rho_{{\mathrm{MoS}}_{2}/\mathrm{metal}}$, $\rho_{{\mathrm{MoS}}_{2}}$, and $\rho_{metal}$ are the charge densities of MoS_2_-metal interfaces, the MoS_2_, and the isolated metal, respectively. To quantitatively describe the charge redistribution in 1L MoS_2_-Au close and vdW interfaces, the plane-averaged electron-density difference Δ*ρ* (z) along the *z* direction is plotted, as shown in Figure 3e and Figure 3f, respectively. We found charge accumulation and depletion at the 1L MoS_2_-Au close and vdW interfaces, which indicate the formation of the interface dipoles. It can be seen that, compared with the case of the 1L MoS_2_-Au close interface (Figure 3e), the charge redistribution of the 1L MoS_2_-Au vdW interface (Figure 3f) is obviously reduced. This implies that the interface dipole at the 1L MoS_2_-Au vdW interface is much smaller than that of 1L MoS_2_-Au close interface. We also plot the plane-averaged electron density difference Δ*ρ* (z) of 1L MoS_2_-Al (Ag, Cu, Pd and Pt) close and vdW interfaces, as displayed in Figure S3 in the supporting information. The same change trend is found for the other metal electrodes in 1L MoS_2_-metal-close and -vdW interfaces. Moreover, it can be found from Table S1 of the supporting information that the amount of charge transfer for 1L MoS_2_-metal vdW interfaces is lower than that for 1L MoS_2_-metal close interfaces, which is consistent with the analysis in Figure 3e,f. Therefore, as compared with the FLP of 1L MoS_2_-metal close interfaces, the reduced FLP strength in 1L MoS_2_-metal vdW interfaces can be attributed to its relatively few MIGS and small interface dipole.

### 3.4. SBH of Multilayer MoS_2_-Metal vdW Interfaces

**Figure 4:**
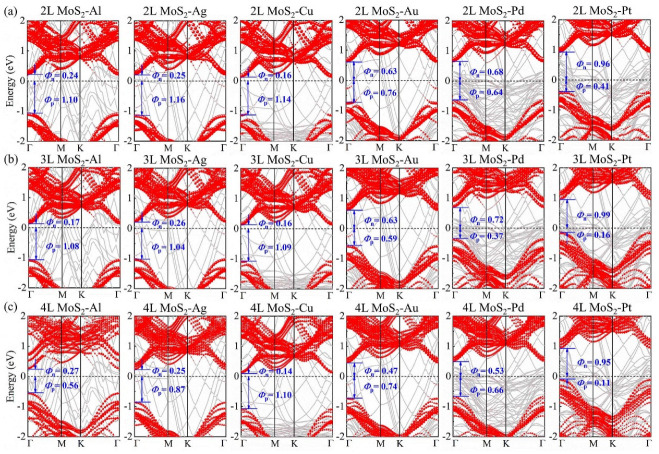
The projected band structures of (**a**) 2L MoS_2_-metal vdW interfaces, (**b**) 3L MoS_2_-metal vdW interfaces, and (**c**) 4L MoS_2_-metal vdW interfaces. The gray curves represent the band structures of MoS_2_-metal interfaces. The red-dotted curves denote the band structures of MoS_2_.

In the following, we further consider the SBH of multilayer (2L, 3L, and 4L) MoS_2_-metal vdW interfaces, and their projected band structures are illustrated in Figure 4. Similar to the case of the 1L MoS_2_-Pt vdW interface, the multilayer MoS_2_-Pt vdW interfaces all present p-type Schottky contacts. As the layer number of MoS_2_ increases, the Fermi level gradually moves close to the VBM of MoS_2_, leading to the decrease of the p-type SBH. Importantly, it can be seen from Figure 4c that a low p-SBH of 0.11 eV can be achieved in the 4L MoS_2_-Pt vdW interface, suggesting the presence of low contact resistance in MoS_2_-based electrical devices.

For monolayer and multilayer MoS_2_-Al (Ag, Cu) vdW interfaces, it can be found from Figure 2g–i and Figure 4 that the Fermi level is closer to the CBM, suggesting the formation of n-type Schottky contacts. In contrast, for MoS_2_-Au (Pd) vdW interfaces we found that their contact types are dependent on the layer number of MoS_2_. Specifically, 1L and 2L MoS_2_-Au vdW interfaces possess the n-type Schottky contacts, and in 3L MoS_2_-Au vdW interface these are changed to the p-type Schottky contact, whereas 4L MoS_2_-Au vdW interface transforms to the n-type Schottky contacts. Similar to the case of the MoS_2_-Au vdW interface, the 1L MoS_2_-Pd vdW interface forms n-type Schottky contacts, and in 2L and 3L MoS_2_-Pd vdW interfaces these transform into p-type Schottky contacts, whereas in the 4L MoS_2_-Pd vdW interface these change back to the n-type Schottky contacts. The transition from n-type Schottky contact to p-type Schottky contact then back to n-type Schottky contact may be correlated with the change trend of the FLP strength in monolayer and multilayer MoS_2_-metal vdW interfaces.

### 3.5. FLP Strength of Multilayer MoS_2_-Metal vdW Interfaces

**Figure 5:**
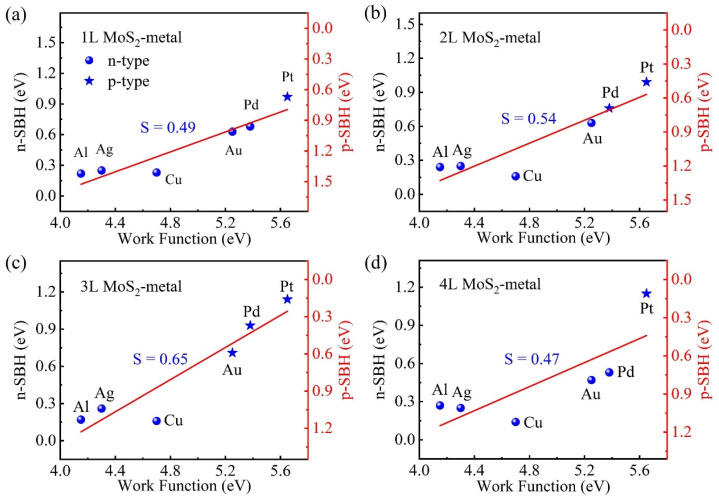
The SBHs versus the metal work functions for (**a**) 1L MoS_2_-metal vdW interfaces, (**b**) 2L MoS_2_-metal vdW interfaces, (**c**) 4L MoS_2_-metal vdW interfaces, and (**d**) 4L MoS_2_-metal vdW interfaces. The pinning factors S are marked in the pictures.

To understand the FLP strength of multilayer MoS_2_-metal vdW interfaces, according to their SBHs, we plot the pinning factors of 2L, 3L, and 4L MoS_2_-metal vdW interfaces, as shown in Figure 5b, Figure 5c and Figure 5d, respectively. For comparison, the pinning factor of 1L MoS_2_-metal vdW interface is also displayed in Figure 5a. It is found that the pinning factors of MoS_2_-metal vdW interfaces are dependent on the layer number of MoS_2_. Specifically, as the layer number of MoS_2_ increases from 1L to 3L, the pinning factor increases from 0.49 to 0.54 to 0.65, indicating that the FLP effect is gradually weakened. In contrast, for the 4L MoS_2_-metal vdW interface the pinning factor decreases to 0.47, suggesting increased FLP. The trend of FLP strength changing as the layer number of MoS_2_ changes can be used to explain the contact type transition in MoS_2_-Au (Pd) vdW interfaces. Taking the MoS_2_-Au vdW interface as an example, as the layer number of MoS_2_ increases to 3L the FLP strength is weakened, and thus the 3L MoS_2_-Au vdW interface is changed to the p-type Schottky contact due to the high work function of Au. When MoS_2_ increases to 4L, the FLP strength is increased, so the 4L MoS_2_-Au vdW interface transforms back to the n-type Schottky contact. The trend of contact type changing as the layer number of MoS_2_ changes is related to the change of FLP strength in the MoS_2_-Au vdW interface, which complies with Schottky–Mott rules.

### 3.6. Layer-Dependent FLP Strength Analysis

**Figure 6:**
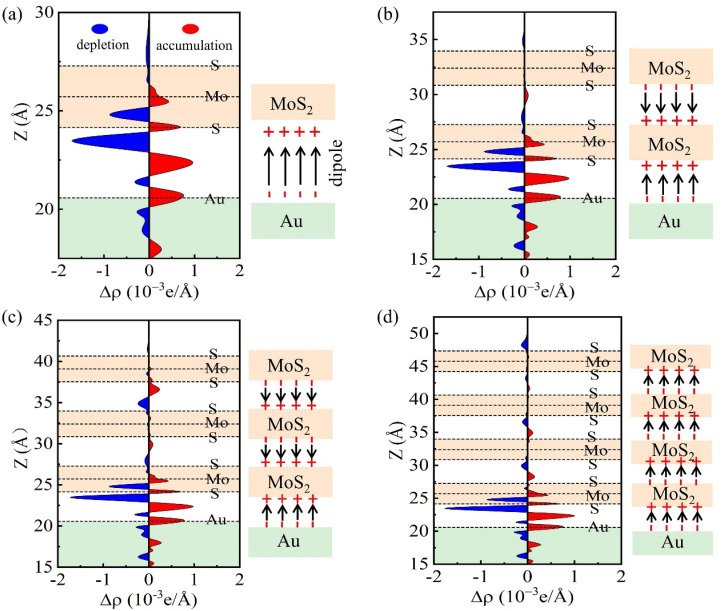
The plane-average charge-density difference (in the left planes) and the schematic illustration of charge transfer (in the right planes) of (**a**) 1L MoS_2_-Au vdW interface, (**b**) 2L MoS_2_-Au vdW interface, (**c**) 3L MoS_2_-Au vdW interface, and (**d**) 4L MoS_2_-Au vdW interface. The arrows represent the direction of interface dipoles.

Next, we analyze the influence of MoS_2_ layer-number on the FLP effect. Taking monolayer and multilayer MoS_2_-Au vdW interfaces as examples, we plotted their plane-average charge-density difference along the z-axis, as illustrated in the left panels of Figure 6. The schematic illustrations of charge transfer at MoS_2_-Au and MoS_2_-MoS_2_ interfaces are displayed in the right panels of Figure 6. It can be found from Figure 6a that the charge accumulates at the Au side and depletes at the MoS_2_ side, indicating that the charge transfers from MoS_2_ to Au, thus creating an interface dipole pointing from Au to MoS_2_ at the 1L MoS_2_-Au vdW interface. For the 2L MoS_2_-Au vdW interface (Figure 6b), the charge redistribution appears at both Au-MoS_2_ and MoS_2_^1st-layer^-MoS_2_^2nd-layer^ interfaces. At the Au-MoS_2_ interface, the dipole direction is the same as in the case of 1L MoS_2_-Au vdW interface. At the MoS_2_^1st-layer^-MoS_2_^2nd-layer^ interface, the charge depletes at the first-layer of MoS_2_ (close to the Au side) and accumulates at the second-layer of MoS_2_ (away from the Au side). The dipole direction at the MoS_2_^1st-layer^-MoS_2_^2nd-layer^ interface is the opposite of that of the Au-MoS_2_ interface, which will weaken the FLP effect at the 2L MoS_2_-Au vdW interface. For the 3L MoS_2_-Au vdW interface (Figure 6c), the charge redistribution occurs at three interfaces: Au-MoS_2_, MoS_2_^1st-layer^-MoS_2_^2nd-layer^, and MoS_2_^2nd-layer^-MoS_2_^3rd-layer^ interfaces. At Au-MoS_2_ and MoS_2_^1st-layer^-MoS_2_^2nd-layer^ interfaces, the dipole directions are the same as that at the 2L MoS_2_-Au vdW interface. At the MoS_2_^2nd-layer^-MoS_2_^3rd-layer^ interface, the dipole direction is same as that of MoS_2_^1st-layer^-MoS_2_^2nd-layer^ interface, which will further weaken the FLP effect. In addition, for the 4L MoS_2_-Au vdW interface (Figure 6d) the dipole directions at MoS_2_-MoS_2_ interfaces are the same as at the Au-MoS_2_ interface, which enhances the FLP effect at the 4L MoS_2_-Au vdW interface. Based on the above discussion, we can conclude that the influence of the MoS_2_ layer-number on the FLP effect can be attributed to charge redistribution at the MoS_2_-metal and MoS_2_-MoS_2_ interfaces.

## 4. Conclusions

In summary, based on DFT calculations we investigate here the FLP effect and the contact types of monolayer and multilayer MoS_2_-metal vdW interfaces. It can be observed that the pinning factor of monolayer MoS_2_-metal vdW interfaces is obviously larger than that of the corresponding close interfaces, indicating that the FLP is weakened at monolayer MoS_2_-metal vdW interfaces. As the layer number of MoS_2_ increases from 1L to 3L, the pinning factor gradually increases, suggesting that the FLP effect is weakened for MoS_2_-metal vdW interfaces. Also, for 4L MoS_2_-metal vdW interfaces the pinning factor decreases to 0.47, suggesting an increase in FLP. The influence of MoS_2_ layer-number on the FLP effect can be attributed to the charge redistribution at the MoS_2_-metal and MoS_2_-MoS_2_ interfaces. In addition, the p-type Schottky contact can be achieved in 1L–4L MoS_2_-Pt, 3L MoS_2_-Au, and 2L and 3L MoS_2_-Pd vdW interfaces, which is related to the change of the FLP effect with the change in layer number of MoS_2_. The p-type Schottky contact is useful for realizing CMOS logic circuits. Our findings demonstrate that the FLP and contact types can be effectively modulated in MoS_2_-metal vdW interfaces dependent on the layer number of MoS_2_, which is helpful for reducing contact resistance and promoting the performance of MoS_2_-based devices.

## Figures and Tables

**Table 1:** Calculated interfacial properties of MoS_2_-metal-close and -vdW interfaces. W_M_ (eV) is the metal work function and δ (%) is the lattice mismatch between MoS_2_ and metal. *d* (Å) and *d*_vdW_ (Å) are the interlayer distances of MoS_2_-metal-close and -vdW interfaces. The binding energies E_b_ (eV) of 1L MoS_2_-metal-close and -vdW interfaces. The n-type and p-type SBHs of MoS_2_-metal-close and -vdW interfaces. The *n* and *p* notations above the SBH represent the n-type and p-type Schottky barrier, respectively.

Metal	W_M_ (eV)	δ (%)	MoS_2_-Metal								
			*d* (Å)	*d*_vdW_ (Å)	E_b_ (eV)		SBH (eV)				
					1L		1L		2L vdW	3L vdW	4L vdW
					Close	vdW	Close	vdW			
Al	4.15	3.35	2.59	3.48	−0.42	−0.29	0.17 *^n^*	0.22 *^n^*	0.24 *^n^*	0.17 *^n^*	0.27 *^n^*
Ag	4.30	4.57	2.64	3.50	−0.59	−0.37	0.11 *^n^*	0.25 *^n^*	0.25 *^n^*	0.26 *^n^*	0.25 *^n^*
Cu	4.70	6.94	2.22	3.46	−0.89	−0.39	0.18 *^n^*	0.15 *^n^*	0.16 *^n^*	0.16 *^n^*	0.14 *^n^*
Au	5.25	4.39	2.81	3.59	−0.50	−0.37	0.27 *^n^*	0.63 *^n^*	0.63 *^n^*	0.59 *^p^*	0.47 *^n^*
Pd	5.38	0.42	2.26	3.59	−0.98	−0.40	0.66 *^n^*	0.68 *^n^*	0.64 *^p^*	0.37 *^p^*	0.53 *^n^*
Pt	5.65	0.45	2.32	3.60	−0.81	−0.43	0.70 *^n^*	0.67 *^p^*	0.41 *^p^*	0.16 *^p^*	0.11 *^p^*

## Data Availability

Data are contained within the article and Supplementary Materials.

## Supplementary Materials

The following supporting information can be downloaded at https://www.mdpi.com/article/10.3390/nano14131075/s1. Figure S1: The band structures of 1L MoS_2_-Cu close interfaces with different supercell match patterns; (a) ($\sqrt{3}\;$×$\;\sqrt{3}$) R30° MoS_2_/(2 × 2) Cu and (b) (4 × 4) MoS_2_/(5 × 5) Cu. Figure S2: The PDOS of 1L MoS2-metal-close and -vdW interfaces; (a) 1L MoS2-Al, (b) 1L MoS2-Ag, (c) 1L MoS2-Cu, (d) 1L MoS2-Au, (e) 1L MoS2-Pd, and (f) 1L MoS2-Pt. Figure S3: The plane average charge density difference Δρ (z) of 1L MoS2-metal-close and -vdW interfaces; (a) 1L MoS2-Al, (b) 1L MoS2-Ag, (c) 1L MoS2-Cu, (d) 1L MoS2-Au, (e) 1L MoS2-Pd, and (f) 1L MoS2-Pt; Table S1: The charge transfer (e) for 1L MoS2-metal-close and -vdW interfaces.
