# Supplementary material for: The Contact Properties of Monolayer and Multilayer MoS2-Metal van der Waals Interfaces
Source: Nanomaterials (Basel). 2024 Jun 24;14(13):1075. doi: 10.3390/nano14131075 (PMC11243427; doi:10.3390/nano14131075)
Supplement: Supplementary file 1 [file nanomaterials-14-01075-s001.zip › nanomaterials-3038062-supplementary.pdf]

Supporting Information

# The Contact Properties of Monolayer and Multilayer $MoS_2$-Metal van der Waals Interfaces

**Xin Pei [1], Xiaohui Hu [1,2,*], Tao Xu [3] and Litao Sun [3]**

1 College of Materials Science and Engineering, Nanjing Tech University, Nanjing 211816, China; 202161103005@njtech.edu.cn
2 Jiangsu Collaborative Innovation Center for Advanced Inorganic Function Composites, Nanjing Tech University, Nanjing 211816, China
3 SEU-FEI Nano-Pico Center, Key Laboratory of MEMS of Ministry of Education, Southeast University, Nanjing 210096, China; xt@seu.edu.cn (T.X.); slt@seu.edu.cn (L.S.)
* Correspondence: xiaohui.hu@njtech.edu.cn

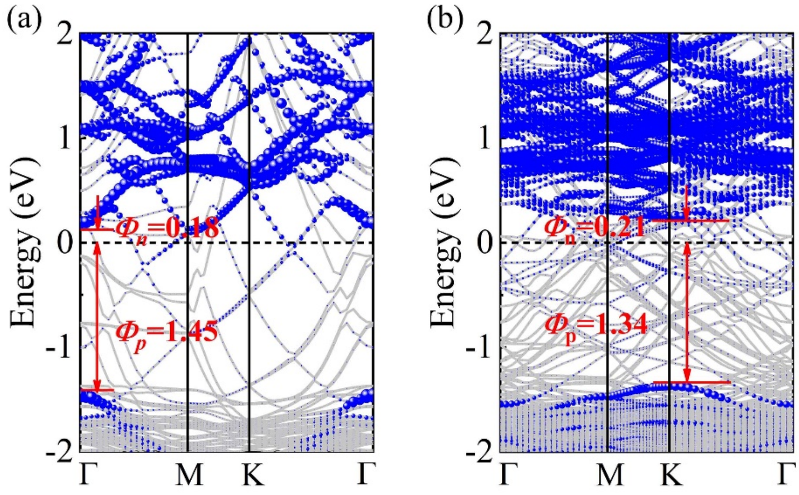


**Figure S1.** The band structures of 1L $MoS_2$-Cu close interfaces with different supercell match patterns. (a) ($\sqrt{3}\times\sqrt{3}$) R30° $MoS_2$/(2×2) Cu and (b) (4×4) $MoS_2$/(5×5) Cu.

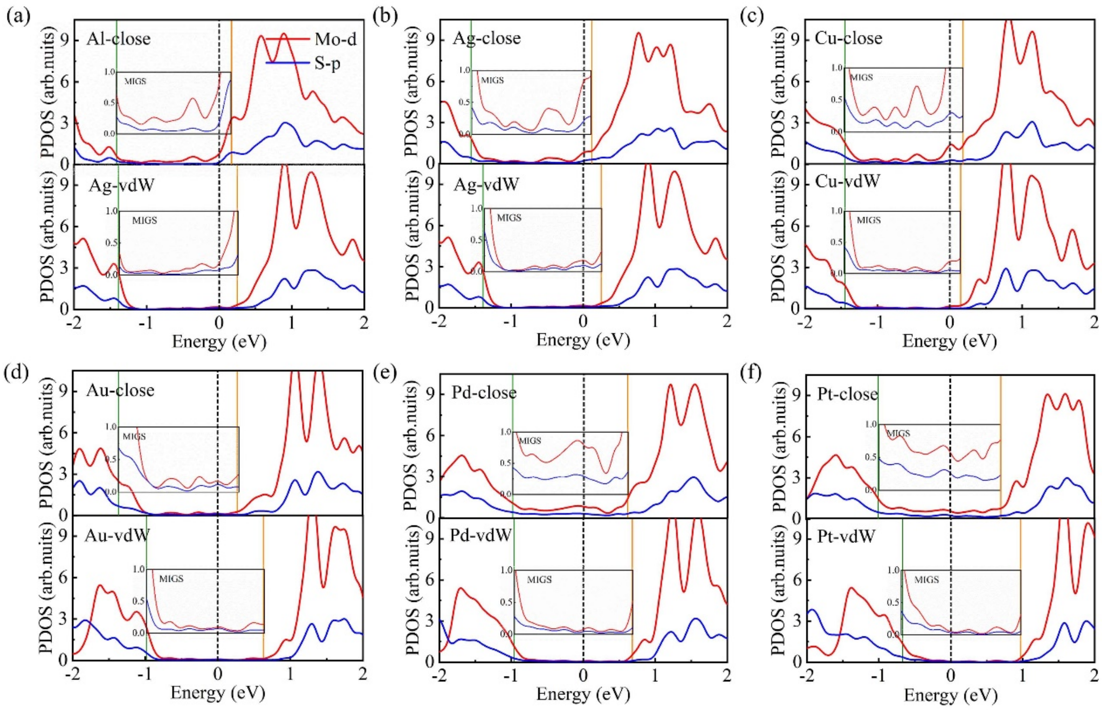


**Figure S2.** The PDOS of 1L $MoS_2$-metal-close and -vdW interfaces. (a) 1L $MoS_2$-Al, (b) 1L $MoS_2$-Ag, (c) 1L $MoS_2$-Cu, (d) 1L $MoS_2$-Au, (e) 1L $MoS_2$-Pd and (f) 1L $MoS_2$-Pt. The magnified PDOS represent the MIGS. Green and orange lines represent the valence band maximum and conduction band minimum, respectively.

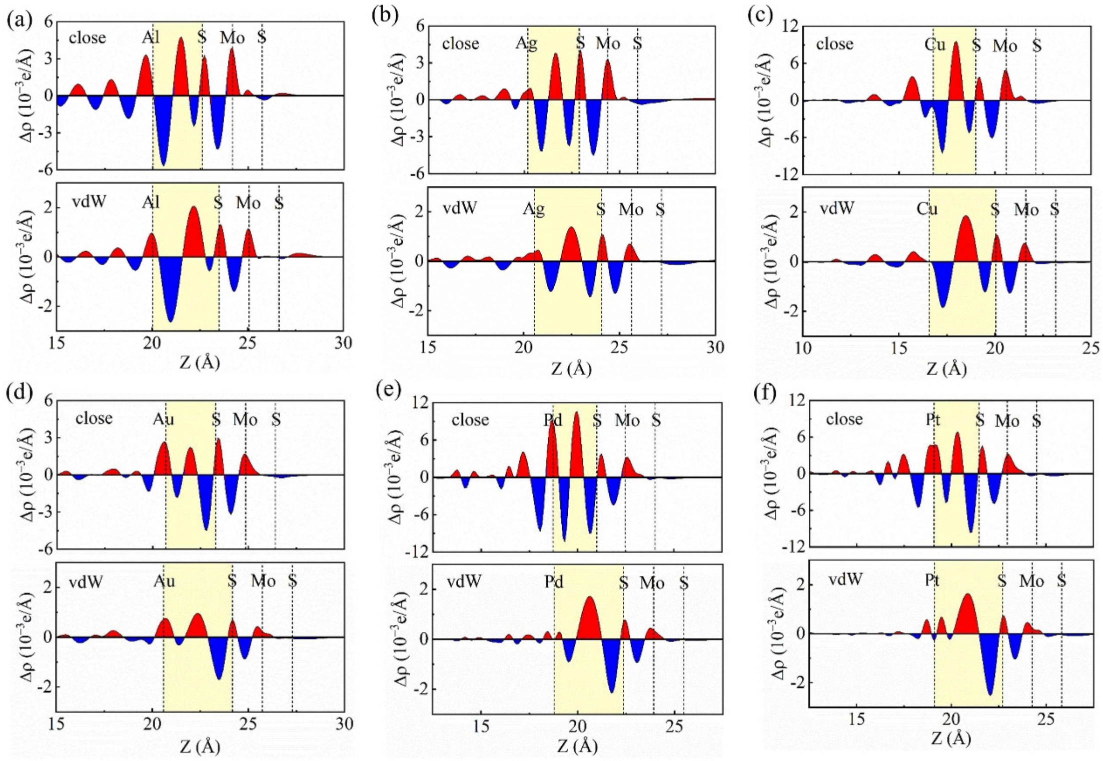

**Figure S3.** The plane average charge density difference $\Delta\rho$ (z) of 1L $MoS_2$-metal-close and -vdW interfaces. (a) 1L $MoS_2$-Al, (b) 1L $MoS_2$-Ag, (c) 1L $MoS_2$-Cu, (d) 1L $MoS_2$-Au, (e) 1L $MoS_2$-Pd and (f) 1L $MoS_2$-Pt. Red and blue regions represent the charge accumulation and depletion, respectively.

**Table S1.** The charge transfer (e) for 1L $MoS_2$-metal-close and -vdW interfaces.

| $MoS_2$-metal | $MoS_2$-Al | $MoS_2$-Ag | $MoS_2$-Cu | $MoS_2$-Au | $MoS_2$-Pd | $MoS_2$-Pt |
|---|---|---|---|---|---|---|
| $MoS_2$-metal close | 0.496 | 0.199 | 0.404 | 0.020 | 0.117 | 0.068 |
| $MoS_2$-metal vdW | 0.230 | 0.097 | 0.148 | 0.004 | 0.041 | -0.012 |
